# Unleashing the transformative power of deliberation with contextual citizens

**DOI:** 10.1098/rsta.2024.0377

**Published:** 2025-11-27

**Authors:** Ariane Lambert-Mogliansky, Irénée Frérot

**Affiliations:** ^1^ Department of Theory, Paris School of Economics, Paris, Île-de-France, France; ^2^ Laboratoire Kastler Brossel, Sorbonne Université, CNRS, ENS-PSL Research University, Collège de France, 4 place Jussieu, 75005 Paris, France

**Keywords:** quantum cognition, thinking frame, deliberations, decision-making, facilitator

## Abstract

In this paper, we investigate deliberation procedures that invite citizens with contextual opinions to explore alternative thinking frames. Contextuality is captured in a simple quantum cognitive model. We show how disagreeing citizens endowed with contextual opinions can reach consensus in a binary collective decision problem with no improvement in their information. A necessary condition is that they are willing to (mentally) experience their fellow citizens’ way of thinking. The diversity of thinking frames is what makes it possible to overcome initial disagreement. Consensus does not emerge spontaneously from deliberations: it requires facilitation.

This article is part of the theme issue ‘Quantum theory and topology in models of decision making (Part 1)’.

## 1. Introduction

Recently, representative democracy has been questioned and is widely perceived as being in crisis in most developed countries. At the same time, more participative forms of democracy are gaining interest [1]. As Niemeyer *et al.* write: ‘Deliberative democracy is now arguably the main theme in both democratic theory and the practice of democratic innovations’ [2]. Theories of collective decision-making are traditionally partitioned into two major fields: those dealing with issues related to the aggregation of diverse preferences (social choice) and those dealing with citizens’ active participation aimed at fostering reciprocal understanding and compromises towards consensus (e.g. [3] for a review).^1^

For participatory democrats from John Stuart Mills to Carole Pateman, the goal of politics is the transformation and education of participants, so that politics is an end in itself [4]. Data from deliberative polls support the hypothesis that people do change their opinion, and this happens not only under the impact of better information [5,6]. Dryzek writes that a ‘defining feature of deliberative democracy is that individuals participating in democratic processes are amenable to changing their minds and their preferences as a result of the reflection induced by deliberation’ [7].

Deliberative democratic theory relies on the principle that ‘outcomes are democratically legitimate if and only if they could be the object of a free and reasoned agreement among equals’ [8]. But how does the process of presenting arguments lead to agreement among equals? Some scholars have argued that reasoned public deliberation lends legitimacy because the proposals that are sustained and survive are simply better in terms of their overall quality. This presumes that there exist some procedure independent criteria of rightness or correctness. Many epistemic democrats hold this view [9,10]. Others have proposed that the very procedure of reasoned public deliberation embodies or manifests core values of basic human morality and political justice, and it forces participants to be attentive towards the common good [8,11–13].^2^ Finally, a number of scholars have argued that reasoned public deliberation complements voting mechanisms by ‘inducing a shared understanding regarding the dimensions of conflict’ [14], which prevents the majority rule from generating majority cycles [5,15,16]). Bohman emphasizes both the transformative and epistemic benefits of confronting a diversity of perspectives in deliberations [17]. Our approach is, in spirit, close to that of Bohman’s. We view the process of deliberation as a procedure that invites citizens to explore alternative perspectives, and we provide a model that links this exploration with the ‘transformative power’ of deliberation that many empirical works emphasize.

The central hypothesis of this paper is in two parts. First, to be able to consider an issue, people have to build a representation of that issue. Building a representation requires selecting a perspective, or equivalently a thinking frame. Second, we assume that some perspectives cannot be considered simultaneously, they are incompatible in the mind of people. This has the crucial implication that no single perspective can aggregate all relevant information: opinions are *contextual*, they depend on the perspectives used to express them. In a close spirit, Niemeyer *et al.* write: ‘Deliberative reasoning as we characterize it, recognises the possibility of identifying the set of relevant considerations, while falling short by failing actively to take all of them into account to capture the complete picture’ [2, p. 347]. Interestingly, it also echoes Socrates who emphasized the irreducible plurality of people’s truth about the world.^3^ To focus on the evolution of opinions due to their contextuality, we consider deliberation exclusively as a process of confronting alternative perspectives with no improvement in information. Kinder writes: ‘frames supply no new information. Rather, by offering a particular perspective, frames *organize—or better reorganize*—information that citizens already have in mind. Frames suggest how politics should be thought about, encouraging citizens to think in particular ways’ [19]. To address the contextuality of opinions, we turn to a widely recognized formal approach that features the co-existence of alternative representations of one and the same object: the Hilbert space model of quantum mechanics (QM). Through the last decades, quantum-like models of contextuality have been developed in social sciences to explain a variety of behavioural anomalies (e.g., [20–22]). We briefly introduce the quantum cognition approach in §2 and then more formally in §3. We emphasize that no prior knowledge of QM or Hilbert space is needed to read the paper.

We consider a setting where a yes or no decision has to be made collectively by majority voting.^4^ Before the vote, the citizens participate in deliberations with the objective of achieving consensus or more realistically, of maximizing the support for the final decision. In this context, the paper addresses two central questions: (1) How can deliberation affect citizens’ voting behaviour when no additional information is provided? (2) How should we structure the process of deliberation to maximize the probability for consensus? A central assumption is that citizens are willing to explore alternative thinking frames before deciding how to vote. These alternative frames can be provided by the citizens themselves or by experts. The procedure is managed by a benevolent facilitator. In the two-person case, when starting from opposite voting intentions, the model predicts that deliberation always achieves some extent of consensus provided that the two citizens do not share the same thinking frame at the outset: diversity of viewpoints is beneficial. The largest chance of consensus is obtained when the citizens’ perspective are maximally uncorrelated.^5^ In the case where the perspectives are two-dimensional, consensus can be reached with probability 3/4 after two rounds. When faced with a population of citizens, the facilitator’s optimal strategy entails a differentiated treatment of the citizens depending on their current opinion. We also find that under some circumstances, overturning the initial majority may turn out to be optimal. In §5, we address some epistemic considerations and briefly discuss the performance of our model with respect to normative and empirical studies.

The main takeaways from our account of thinking frames in the analysis of deliberation are the following : (i) the diversity of thinking frames among citizens not only is no obstacle but is a necessary condition for deliberation to deliver consensus; (ii) deliberation can exhibit a transformative power that hinges on a willingness of participants to explore alternative thinking frames; and (iii) well-designed procedures monitored by a facilitator have a significant potential to help people reach consensus.

This paper contributes to the literature on the value of pre-voting deliberation by providing a formalization of opinion formation, appealing to the intrinsic contextuality of opinions. Most formal approaches to deliberation belong to the epistemic tradition, which postulates a single (common) correct decision. Among the most recent ones, Dietrich & Spiekermann introduce behavioural features to the information theoretic approach (e.g., [24]). The other strand of the formalized literature is game-theoretic. It emphasizes incentives to share or withhold information (e.g., [25]). The quantum (contextuality) revolution has instead recast the issue of objective truth and knowledge, as witnessed by the wealth of the epistemological literature over the last century (e.g. [26]). Our approach, based on the most standard formalization of intrinsic contextuality, is closely related to Bohman’s experiential perspective approach in [17]. He emphasizes the transformative and epistemic benefits of confronting a diversity of perspectives in deliberations. We provide a formal description for this transformation and derive optimal procedures managed by a facilitator. Because we preclude improvement in information, our approach is complementary to classical epistemic approaches. But since intrinsic contextuality precludes the uniqueness of truth, it brings us close to procedural approaches to deliberation [27] which recognize a value to deliberation in terms of realizing basic values of democracy. Our results also contribute with new results regarding the tension between efficiency and legitimacy within the process of deliberations itself [28]. Finally, our analysis produces a novel insight into the value of diversity, namely, that it contributes to overcoming disagreement, which is consistent with the (empirical) literature on teamwork (e.g. [29]). The rest of the paper is organized as follows. In §2, we provide a brief account of the quantum cognition approach. In §3, we present the model. The analysis is developed in §4 where our two main results are formulated. We conclude, in §5, with some remarks.

## 2. Contextuality in social sciences

**Figure 1.**
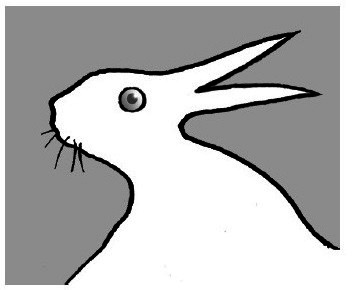
What do you see?

Human beings often face difficulties in grasping complex issues. We consider reality by focusing on one perspective at a time and have trouble combining perspectives *in a stable way*. This inability to seize reality in its full richness suggests that the process of developing an understanding of the world may not look like a puzzle that is assembled progressively. Instead, the human mind may exhibit structural ‘limitations’ in terms of the incompatibility of perspectives. The psychological process of learning is then better described as an explorative and transformative journey through different landscapes. Ambiguous pictures such as figure 1 provide a suggestive illustration of the kind of incompatibility we have in mind. You may see a duck or a rabbit. You may oscillate between the two. Both are correct, but you cannot see both simultaneously.^6^

At first sight, it may seem quite artificial to turn to QM when investigating human behavioural phenomena. However, the founders of QM, including Bohr and Heisenberg, recognized an essential similarity between the two fields.^7^ In both fields, the object of investigation cannot always be separated from the process of investigation. QM and, in particular, its mathematiformalism, was developed to respond to a general epistemological challenge: how can one study an object that is being modified in an uncontrollable way in the process of measurement of its properties? QM is a general paradigm for intrinsic contextuality (i.e. non-separability between the object and the operation of investigation). It is therefore fully legitimate to explore the value of the mathematical formalism of QM in the study of human behavioural phenomena—without any reference to physics.

The quantum paradigm has been proposed in decision theory and psychology to describe preferences, beliefs, attitudes, judgments and opinions (e.g. [20–22,33–35] among others). Quantum cognition has allowed providing a unified framework that accommodates a wide variety of so-called behavioural anomalies: preference reversal, disjunction effect, cognitive dissonance, framing effects, order effects, among others (e.g. [35], and for empirical applications [36,37]).

In this paper, our starting point is that the representation of the world is a mental object that may exhibit non-classical features, and we derive some implications for the dynamics of opinion formation in the process of deliberation. The most important element that we borrow from QM is the notion of Bohr-complementarity, applied here to mental perspectives.^8^ In line with quantum cognition, we propose that Bohr-complementarity of perspectives captures the cognitive limitations that are responsible for our difficulties to synthesize information from different perspectives into a single stable picture. Just as in QM, the system (here our mental picture) makes discrete jumps when attempting to find a determinate value along distinct incompatible perspectives, so that the final picture depends on the path of exploration.

Recently, theoretical and experimental applications to persuasion have shown how fruitful this approach could be [37–39]. This paper is in continuation with those works. A citizen’s opinion is formalized as a quantum object characterized by its state vector. Alternative thinking frames are modeled as alternative bases, representing incompatible perspectives, on the (mental representation of the) decision issue. Deliberation amounts to a sequence of measurements (probing arguments). Measurements move the opinion state in a non-deterministic way that reflects the correlations between the perspectives (the thinking frames). With this formal description of the process of deliberation, we investigate procedures that satisfy some requirements put forward in the literature on democratic deliberations.

We emphasize that the quantum cognition approach does not assume a quantum physical nature of the determinants of our opinions. Neither do we dwell on the psychology or neurology of the transformation of belief/opinion and preferences.^9^ A presumption is that the correlations between the perspectives that structure the mind exhibit some extent of regularity across individuals. The quantification of such correlations remains an empirical question open to future work.^10^

## 3. Model

### (a) Basic structure

We formulate our model of deliberation in terms of a communication protocol. We consider a set Ω={1,2,…,N} of deliberator-citizens (she), one facilitator (he) and a pool of experts. We are interested in deliberations aimed at influencing citizens’ vote over uncertain options, which we model as quantum lotteries, following [33].^11^ The formal model shares significant features with the quantum persuasion model in [38,39].

Throughout the paper, we shall illustrate the concepts with the following example:

*A community needs to decide whether to introduce an Individual Carbon Budget* (*ICB*) *scheme or not. Various aspects are of relevance to citizens: environmental efficacy, impact on individual liberties, legal feasibility, etc. The community members deliberate before deciding by majority vote*.

### (b) Opinions and perspectives

A citizen’s opinion, e.g. ‘I think ICB is a good way to reduce CO_${,}^{2}$_ emissions’, is a mental object that we model as a quantum-like system. The description of a quantum system starts with the definition of a Hilbert space H which we take over the field ℝ of real numbers and of finite dimension n.^12^

*Opinion state*

Using Dirac’s notation, a citizen is characterized by her opinion state, a vector |ψ⟩∈H and her thinking frame or perspective denoted P (see below for formal definition). Let $P_{\left|\psi \right\rangle }$ be the orthogonal projector onto |ψ⟩, $P_{\left|\psi \right\rangle }=|\psi \rangle \langle \psi |.$^13^ Using the trace operator, we have $\text{Tr}(P_{\left|\psi \right\rangle })=\text{Tr}(|\psi \rangle \langle \psi |)=\langle \psi ||\psi \rangle =1$. States corresponding to one-dimensional projectors are called pure states.^14^ In this paper, we shall only be dealing with pure states, and we often use the operator formulation to express states. The non-negativity of the operator $P_{\left|\psi \right\rangle }$ is analogous to the non-negativity of a probability measure, and trace 1 analogous to the sum of probabilities which equals 1. This means that an opinion state is formally identical to a (subjective) belief state.

*Perspectives*

The formal account of thinking frames (or perspectives; we use the terms interchangeably) is a key building block of our theory. It is intimately linked with our cognitive assumption below. Our model associates the citizens (and the experts) each with a perspective. In the ICB example, one citizen may be endowed with an environmental perspective, another with a libertarian one. A perspective


(3.1) $$\begin{array}{lcr} & P=\left(P_{1},...,P_{n}\right) & \end{array}$$

is a collection of pairwise orthogonal one-dimensional projectors (corresponding to the possible opinion states in P), that is, $P_{i}^{2}=P_{i}$, and $\text{Tr}(P_{i}P_{j})=0$ if i≠j, and $\sum_{i=1}^{n}P_{i}=E$, where E is the identity operator on H. A perspective P is incompatible with perspective $Q=\left(Q_{1},\ldots ,Q_{n}\right)$ if $P_{i}Q_{j}\neq Q_{j}P_{i}$ for some of the i,j. The correlation between perspectives is captured by the trace of the products $\text{Tr}\left(P_{i}Q_{j}\right)$. The incompatibility of Q and P is then equivalent to $\text{Tr}\left(P_{i}Q_{j}\right)\notin \left\{0,1\right\}$ for some of the i,j. Geometrically, two incompatible perspectives define two non-collinear orthonormal bases in the Hilbert space. The concept of perspective allows capturing our central cognitive assumption.

**Assumption 1**

(i) *Citizens cannot address reality immediately. They need to build a representation of the voting issue using a thinking frame*;
(ii) *Citizens cannot resort to a ‘super frame’ that would aggregate all relevant aspects.*

Assumption 1 implies that, for citizens, the voting issue admits several equally valid but incompatible thinking frames, the *perspectives*. Incompatible perspectives are complementary in the description of the issue, but they cannot be considered simultaneously, only alternatively. It is assumption 1 that leads us to use the formal framework of QM, as a mathematical tool with such built-in complementarity. Incompatible perspectives are formally similar to Bohr-complementary observables in quantum physics (e.g. the position and velocity of a particle).

*Probing a perspective*

Probing a perspective is analogous to performing a measurement in QM. It is an operation where a citizen in opinion state |ψ⟩ subjects herself to a perspective $P=\left(P_{1},\ldots ,P_{n}\right)$. This results in one of the possible outcomes (1,..,n) with respective probabilities


(3.2) $$p_{i}=\langle \psi |P_{i}|\psi \rangle =\text{Tr}(|\psi \rangle \langle \psi |P_{i})=\text{Tr}(P_{\psi }P_{i}),$$

for i=1,..,n. In deliberation terms, it corresponds to questioning oneself in terms of perspective P. For instance, in the environmental perspective: ‘Do I think that ICB is an efficient way to reduce CO_${,}^{2}$_ emissions or not?’ We focus exclusively on *complete measurements*, that is, on probing operations that fully resolve uncertainty regarding the perspective that is being probed. The outcome of probing a perspective is one of the labels i∈{1,…,n}, here corresponding, e.g., to the opinion: ‘I believe ICB is efficient to reduce CO2 emissions’.

When the probing operation yields outcome i, the opinion state $P_{\psi }$ moves into the (revised) opinion state $P_{i}$. Hence, probing the same perspective twice in a row yields the same outcome since $\text{Tr}(P_{i}P_{i})=1$. The opinion state does not change. An important feature that we want to emphasize is that the expected revised opinion $P_{\psi }^{ex}=\sum_{i=1}^{n}p_{i}P_{i}=\sum_{i=1}^{n}P_{i}P_{\psi }P_{i}$ is generally different from the initial opinion state $P_{\psi }$. That is, although the opinion state has the structure of a probability distribution, the revised opinion in the quantum formalism is *not* subject to Bayesian plausibility, as noted in [39]. This feature plays an important role in the analysis.

**Figure 2.**
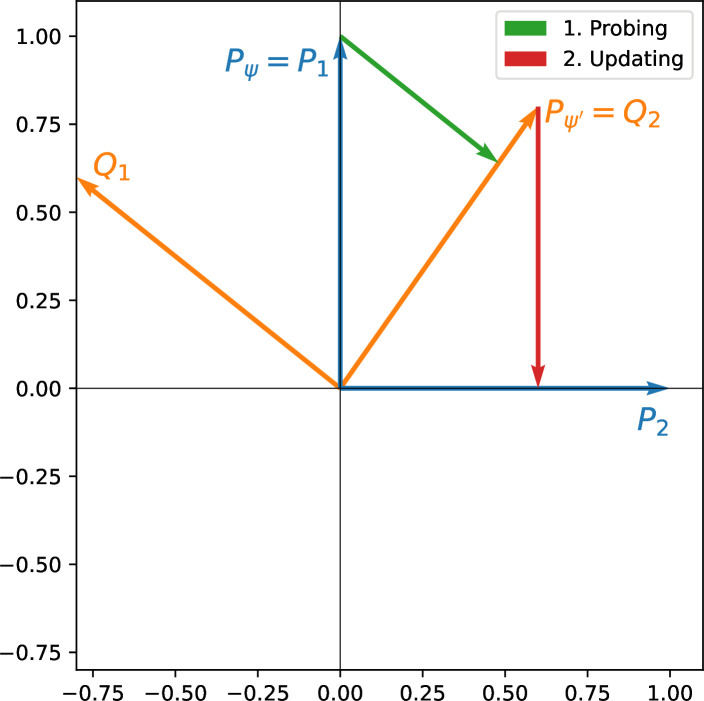
Probing perspectives

For the sake of illustration, consider the two-dimensional example in figure 2. Our citizen is initially characterized by her thinking frame P (say the environmental) and opinion state $P_{\psi }=P_{1}$ (e.g. corresponding to ‘ICB is an efficient tool to reduce CO_2_ emissions’). Probing the *alternative* (i.e. incompatible) perspective $Q=\left(Q_{1},Q_{2}\right)$ (e.g. the libertarian one that focuses on individual liberties), which we refer to as *challenging one’s opinion,* results in a new state $Q_{j}$, with probability $\text{Tr}(P_{i}Q_{j})$. Say the result is state $P_{\psi^{\prime }}=Q_{2}$. When the citizen updates her opinion, i.e. probes P anew (to be able to evaluate the voting options, see below), we have $\text{Tr}\left(Q_{2}P_{2}\right)>0$. So with positive probability, our citizen has changed opinion into $P_{2}$: she now believes that ICB schemes are environmentally useless. This is the crucial property that generates a potential for opinions to evolve without additional information.

### (c) Utility and voting

Voting is a binary, yes or no, choice. Citizens are endowed with preferences that allow them to evaluate the (expected) utility value of the two options given their individual opinion state.

**Assumption 2**

A *citizen can only attribute utility value to voting options in one of the eigenstates of her own frame, i.e. in some state* $P_{\psi }=P_{i}$ *where* $P=\left(P_{1},\ldots ,P_{n}\right)$ *is the citizen’s own perspective*.

Assumption 2 captures a central feature of thinking frames. The own frame is an essential part of a citizen’s identity; it is the language in which she can formulate the value of options and make decisions. Citizens can explore other perspectives and adopt any possible opinion. However, an opinion state can guide voting *only* when formulated in terms of the own perspective. Formally, consider a citizen with perspective $P=\left(P_{1},...,P_{n}\right)$. Her utility function is represented by a tuple $\left\{u_{1}^{Y},..,u_{n}^{Y},u_{1}^{N},...,u_{n}^{N}\right\}$ which associates a real number $u_{i}^{Y}$ to voting option yes, respectively, $u_{i}^{N}$ to voting option no, for each possible opinion state $P_{i}$. For any arbitrary opinion state $P_{\psi }$, we formulate the expected utility for the two voting options as a ‘quantum lottery’ [33], with the probability for each of the states $P_{i}$ given by $\text{Tr}(P_{\psi }P_{i})$. The formula for the expected utility is


(3.3) $$EU\left(Y;P_{\psi }\right)=\sum_{i=1}^{n}Tr\left(P_{\psi }P_{i}\right)u_{i}^{Y},$$

and similarly $\mathrm{EU}\left(N;P_{\psi }\right)=\sum_{i=1}^{n}\mathrm{Tr}\left(P_{\psi }P_{i}\right)u_{i}^{N}$. The expression in equation (3.3) assumes that citizens are able to compute $\text{Tr}\left(P_{\psi }P_{i}\right)$ for any $P_{\psi }$. This is very demanding. In particular, it requires that citizens know the correlations between all perspectives alternative to their own. We do not make that assumption. Instead, whenever in $P_{\psi }\notin P$, the citizen has to update her opinion state (by probing their own thinking frame) before voting.

**Assumption 3**

*A citizen is endowed with a thinking frame that allows separating between voting options : for each citizen with perspective* P*,* $\exists P_{i},P_{j}$ *two states, such that* $u_{i}^{Y}\geq u_{i}^{N}$ *and* $P_{j};u_{j}^{N}>u_{j}^{Y}$.

Assumption 3 excludes citizens whose voting decision is fixed and therefore cannot be affected by deliberations. The voting behaviour is then most simple: by assumption 2, the citizen must be in one of her perspective’s eigenstates, say $P_{i}$; then if $u_{i}^{Y}\geq u_{i}^{N}$, she casts a yes vote; otherwise she casts a no vote. Hence, we assume that voting is sincere (non-strategic).^15^ The winning option is the one that obtains the largest number of votes in the population of deliberating citizens. In case of a tie, at the end of the deliberations, a random draw determines the outcome.

### (d) Deliberation

Deliberation is modelled in terms of a multiple-round communication protocol monitored by the facilitator. The citizens participating in deliberations are assumed to be open-minded, i.e. they are willing to engage in real mental experiences. This captures a leitmotiv in the literature on deliberation, which recognizes the value of reciprocal respect and goodwill.

**Assumption 4**

*Citizens taking part in deliberation follow the recommendations of the facilitator*.

The facilitator’s recommendations are of two types. He may invite a citizen or an expert to present an opinion, and he may invite a subgroup of citizens to be active, i.e. to probe some perspective. When hearing an opinion from P, an active citizen explores that perspective by thinking in its terms, and determines which opinion she agrees with.^16^ After challenging her opinion by probing P, citizen j updates her opinion by reassessing her position in her own perspective $P^{j}$ so as to be able to evaluate the utility value of the voting options, as described above. All other citizens are invited to remain ‘passive’, i.e*.* they only listen to the argument but do not explore the corresponding perspective and thus do not change opinion.^17^.

In this paper, we are not addressing possible incentive issues related to these operations. By assumption 4, citizens follow the recommendations, and we assume benevolence from the side of the facilitator, as captured in his objective function below.^18^

*The facilitator strategy*

In each round, the facilitator, who is the sole true decision-maker, makes two choices: a perspective P (chosen among the citizens’ and experts’ perspectives) and ω⊂Ω, a subset of citizens who are invited to be active. The facilitator is benevolent, which is fully captured in his objective function: he maximizes the support for the most supported voting option, which we call the *score* of that option*.*^19^ A crucial remark is that the vector of opinion states, at time t, $P_{\psi }^{t}=\left(P_{\psi_{1}}^{t},\ldots P_{\psi_{N}}^{t}\right)$ captures all relevant information about history up to time t. This follows from the fact that the probabilities for opinion change when probing perspective $P=\left(P_{1},\ldots P_{n}\right)$ for a citizen in state $P_{\psi }$ is given by $\text{Tr}(P_{\psi }P_{i})$, i.e., they *only* depend on her current state of opinion—not on previous history.^20^ The vector $P_{\psi }^{t}$ corresponds to the opinion states *at the beginning* of round t (see the ‘timing’ below). Finally, we restrict our attention to strategies that maximize the expected score *in each round.*^21^ A strategy for the facilitator is a function from a vector of opinions $P_{\psi }^{t}$ to a pair (P,ω) including a perspective P and a subset ω of citizens: $P_{\psi }^{t}\to \mathbb{P}\times \left(2^{N}-1\right)$, where ℙ is the set of all perspectives and $2^{N}-1$ counts the number of non-empty sets of Ω={1,…,N}.

We make the following informational assumption:

**Assumption 5**

*The facilitator has knowledge about all the correlations between all the perspectives, i.e.,* $\text{Tr}(P_{{,}_{j}}Q_{i})$ *for all* i,j=1,..n *and for all* P,Q*.*

The facilitator has an informational advantage when compared with the citizens in terms of knowing the full structure of the opinion state space. He is aware of all possible perspectives and how they correlate to each other. This is the critical resource that allows him to optimize the deliberation process. He also has access to a pool of experts who can present arguments from any possible perspective $P^{e}$. Experts are simply ‘tools’ that the facilitator can call upon whenever he wants. However, since the spirit of deliberation is to give voice to citizens, we shall give particular attention to what can be achieved without appealing to experts.

## 4. Analysis

We start the analysis with deliberations between two citizens. This allows deriving our main results, which we later extend to deliberations involving a group of citizens.

### (a) Deliberation with two citizens

We have two citizens, Alice and Bob who face a binary collective decision about, e.g. the introduction of an Individual Carbon Budget scheme. There exist two relevant aspects: environmental efficacy and individual liberty. These two aspects cannot be considered simultaneously by our citizens, they are assumed to be incompatible in the mind.

The perspectives are n-dimensional: the larger, n, the finer the characterization of the opinions. In terms of our example, the environmental perspective can have several possible values (each associated with its own eigenstate), e.g. ICB is the best solution for reducing greenhouse gas (GHG), ICB is one among the best solutions to reduce GHG, ICB is a good solution etc; until ICB is worthless to reduce GHG. For the ease of presentation, we shall focus on the case n=2, i.e. there are only two possible values for the opinion in each perspective. This simple case allows establishing some central results (for more general results, see our article [40]).

The citizens are endowed with

(i) An opinion state, which is denoted *A* for Alice’s and *B* for Bob’s ;
(ii) An own perspective, denoted $A=(A_{1},A_{2})$ for Alice's (environmental) perspective and $B=(B_{1},B_{2})$ for Bob's (libertarian) perspective ;
(iii) A set of utility values associated with the own perspective's eigenstates: $\{u_{A_{1}}^{Y},u_{A_{2}}^{Y},u_{A_{1}}^{N},$ $u_{A_{2}}^{N}\},u_{A_{i}}^{Y}$, is the utility value for Alice corresponding to the yes vote in opinion state A_i_, and $u_{A_{i}}^{N}$ the utility value corresponding to the no vote. We similarly define $u_{B_{j}}^{Y},u_{B_{j}}^{N},j=1,2$ for Bob.

*Deliberation protocol*. Before Bob and Alice start deliberating, they update their opinion, that is, they probe their own perspective.^22^ The initial individual opinion states are therefore always eigenstates of the own perspective: some $A_{i}$ for Alice and some $B_{j}$ for Bob. We consider a process where the facilitator does not appeal to experts in the first two rounds (which we call the ‘voice phase’). Since we only have two citizens, the set of active citizens $\omega^{t}$ boils down to the one citizen not invited to present her argument in round t.

*Timing* t=0. The facilitator asks for initial voting intentions. If Alice and Bob agree, the procedure requires no deliberation. If they disagree, the first round starts.

The facilitator asks for initial voting intentions. If Alice and Bob agree, the procedure requires no deliberation. If they disagree, the first round starts.

*Round t = 1*

— The facilitator lets a random draw determine who will present his/her argument first, say Alice;
— Alice argues for Bob in her terms (i.e., in terms of her thinking frame) and Bob responds by probing Alice’s perspective;
— Bob updates his opinion by probing his own perspective.

If disagreement on voting remains, a second round starts:

*Round t = 2*

— Bob exposes his opinion and Alice responds by probing Bob’s perspective;
— Alice updates her opinion by probing her own perspective;

Out of the resulting opinion states, if they still do not agree, a new round starts, appealing to an expert and so on until t=T.

If disagreement persists at t=T, the decision regarding ICB is determined by a random device.

Notice that Alice’s and Bob’s opinions are two separate quantum-like systems. The state of the global system at time step t is denoted as ${\text{P}}_{\psi }^{t}=(P_{\psi_{A}}^{t},P_{\psi_{B}}^{t})$.^23^ When each citizen’s opinion state is an eigenstate of the own perspective (which is required to vote by assumption 2), ${\text{P}}_{\psi }^{t}\in \left\{\left(A_{1},B_{1}),(A_{1},B_{2}),(A_{2},B_{1}),(A_{2},B_{2}\right)\right\}$. By virtue of assumption 3, we define $\left(A_{1},B_{1}\right)$ as the opinion state leading to a consensual yes vote; and $\left(A_{2},B_{2}\right)$ as the opinion state leading to a consensual no vote.^24^ In both consensual states, the facilitator’s score is maximal and equal to 2. When the current opinion state ${\text{P}}_{\psi }^{t}\notin \left\{\left(A_{1},B_{1}),(A_{2},B_{2}\right)\right\}$, the facilitator needs one of the citizens to change opinion. Maximizing the expected score is equivalent to maximizing the probability that this happens. In the two-dimensional case, the probability for Alice or Bob to change opinion when probing the other’s perspective is entirely governed by a single parameter: $x=\mathrm{Tr}(A_{1}B_{1})$. Indeed, as $A_{1}+A_{2}=E=B_{1}+B_{2}$, with E the identity operator, we have $\mathrm{Tr}(A_{2}B_{1})=\mathrm{Tr}(A_{1}B_{2})=1-x$, and $\mathrm{Tr}(A_{2}B_{2})=x$.

We consider deliberations starting from initial disagreement, say Alice and Bob have respective opinion states $A_{1}$ and $B_{2}$. From the facilitator’s point of view, the two consensus states $\left(A_{1},B_{1}\right)$ and $\left(A_{2},B_{2}\right)$ are fully symmetric. Let the initial random draw give Alice the initiative. The facilitator invites her to present her argument, while Bob is invited to probe Alice’s perspective. Bob’s opinion state moves into $A_{1}$ with probability $\mathrm{Tr}(B_{2}A_{1})=\left(1-x\right)$, and into $A_{2}$ with probability $\mathrm{Tr}(B_{2}A_{2})=x$. Bob thereafter updates his opinion by probing his own perspective. The probability for reaching consensus is equal to the probability that Bob’s new opinion state is $B_{1}$ (instead of the initial $B_{2}$):


(4.1)prob(B2→B1)=Tr(B2A1)Tr(A1B1)+Tr(B2A2)Tr(A2B1)(4.2)=2x(1−x) .

Since only Bob’s opinion has been challenged, the other consensual state (i.e. $\left(A_{2},B_{2}\right)$) cannot have emerged.

*Maximally uncorrelated perspectives.* Generally, perspectives A and B are said to be maximally uncorrelated when $\mathrm{Tr}(B_{i}A_{j})=1/n$ for all i,j (with n=2 in the present context).^25^ When Bob probes the A perspective, he has equal chance (1/2) to move into any of the states $A_{i}$. As Bob next updates his opinion by probing the B perspective, he reaches any of the opinion states $B_{j}$ with equal probability. Effectively, Bob’s initial opinion state has been completely randomized by the (intermediate) operation of probing of Alice’s (maximally uncorrelated) perspective.

The second round following disagreement proceeds similarly, generating consensus in state $A_{2}B_{2}$ with the same probability 2x(1−x). Hence, the probability of reaching consensus after the first two rounds is 2x(1−x)+(1−2x(1−x))2x(1−x)=4x(1−x)[1−x(1−x)].

We have the following.

**Proposition 1**

*Starting from a disagreement on voting between two citizens:*

(i) *Fact-free deliberation between two citizens who share the same (formal) perspective*^*26*^ *has no impact on their opinions;*

(ii) *With distinct perspectives, consensus is reached with strictly positive probability after a first round;*

(iii) *The probability for consensus is largest when the perspectives are maximally uncorrelated, it reaches after two rounds*.

*Proof*. (i) When the two perspectives are fully correlated, we have $\mathrm{Tr}(A_{i}B_{j})\in \left\{0,1\right\}$*,* the opinion states are either equal ($A_{1}=B_{1}$ and $A_{2}=B_{2}$) or orthogonal ($A_{1}=B_{2}$ and $A_{2}=B_{1}$). Bob’s and Alice’s perspectives are formally indistinguishable. In this case, no transition $B_{1}\to A_{i}\to B_{2}$ or $B_{2}\to A_{i}\to B_{1}$ can ever occur by probing the A perspective, which has no impact whatsoever on Bob’s opinion state: 2x(1−x)=0. The initial disagreement cannot be overcome through deliberations.

(ii) First, note that from the point of view of the facilitator, the two consensus states are fully symmetric $\mathrm{prob}(B_{2}\to B_{1})=\mathrm{prob}(A_{1}\to A_{2})=2x\left(1-x)\right)$ so a random draw is optimal for the facilitator. The result from proposition 1(ii) follows from equation (4.2), which shows that the probability of reaching consensus is strictly positive whenever 0<x<1, that is, when perspectives A and B are distinct.

(iii) The probability for consensus in the first round is maximal at x=1/2 where $\frac{\partial }{\partial x}[2\left(1-x\right)x]=0,x\in \left[0,1\right]$. The two perspectives are then maximally uncorrelated. The total probability for success after the second round, conditional on failure in the first round, is 4x(1−x)[1−2x(1−x)], which reaches its maximum equal to 3/4 for uncorrelated perspective as well, i.e. x=1/2.∎

Proposition 1(i) is quite remarkable because it shows that starting from disagreement, sharing the same thinking frame is an obstacle to achieving consensus. Indeed, within a common thinking frame, citizens can only update their opinion in response to new information (by Bayesian updating) which we preclude in this paper. However, when citizens are endowed with distinct perspectives, new opportunities for opinion to evolve arise. By actively exploring a perspective incompatible with one’s own, intrinsic contextuality reveals its transformative power. Exploring an alternative perspective changes the opinion state because the possible outcomes of that operation do not exist in the own perspective. The opinion state is forced into a new state. This result about the value of diversity is truly novel and a main contribution of this paper. It is important at this point to emphasize that our deliberation protocol demands a true mental experience, i.e. sincerely ‘putting oneself in someone else’s shoes’—the probing operation. This means that Alice sincerely recognizes that Bob has a point in bringing up individual liberties, and similarly for Bob.

Proposition 1(ii) quantifies how the diversity of perspectives allows opinions to evolve towards consensus. The weaker the correlation between perspectivesx→1/2, the larger the impact of the probing operation. The intuition is that the more closely related the perspectives, the more likely that probing Alice’s frame takes Bob to an opinion state (in A) close to his initial state (when $B_{2}$ is close to $A_{1}$, $\text{Tr}(B_{2}A_{1})$ is large). When probing his own perspective anew, he is most likely confirmed in his initial opinion. Similarly for Alice, so disagreement is more likely to persist. Nevertheless, with some positive probability, one of the two citizens will have changed his or her mind, which implies consensus on voting. Interestingly, the result that uncorrelated perspectives give the best chance for deliberation to achieve consensus, reminds us of a result in quantum persuasion [39], where the authors show that distraction understood as bringing attention to a perspective uncorrelated to the targeted belief state is part of an efficient strategy to persuade a person.

Proposition 1(iii) says, without surprise, that starting from dissensus, additional rounds following failure to reach agreement increase the probability for consensus. While a single round can already achieve consensus with probability 2(1−x)x, with two rounds and maximally uncorrelated perspective case (x=1/2), we reach consensus in 75% of the cases. Of course, we do not expect citizens to repeat the same argument from round to round. When the citizens are short of arguments, it is definitely time for the facilitator to call on experts with suitable alternative perspectives.

**Corollary 1**

*The first-moving citizen has a larger chance to see consensus on her initial voting preferences than the one who moves second.*

*Proof*. As earlier noted that the chance of reaching consensus is the same whoever is selected first: $\text{prob}\left[(A_{1},B_{2})\to (A_{1},B_{1})\right]=2x\left(1-x\right)=\text{prob}\left[(A_{1},B_{2})\to (A_{2},B_{2})\right]$. But since the second case is only possible in the case of failure in the first round, it has less chance to be selected in the vote.∎

While the procedure gives more chance to the first selected citizen, the introduction of a random draw restores the equality of chance between the citizens.

**Corollary 2**

*When citizens’ perspectives are correlated, relying on an expert with a perspective uncorrelated to the current round’s active citizen’s perspective, increases the chance for reaching consensus in any given round*.

This follows from proposition 1(ii). When Alice and Bob have correlated perspectives, the probability for reaching consensus when probing each other’s perspective is lower than 75% after two rounds. The score is maximized when only experts are presenting arguments belonging to a perspective maximally uncorrelated with the active citizen’s.

Corollary 2 implies that there exists a tension between the desire to have citizens probing each other’s perspectives and the objective to maximize the score. This is not surprising given proposition 1(ii). To preserve the democratic character of deliberation, and to give voice to citizens, a mixture of citizen arguments and expert arguments can be chosen, at the cost of some delay in reaching consensus, however. As proposed above, deliberations can start with a voice phase where citizens present their own views, and, if it fails, proceed with an expert phase.

### (b) Deliberation in a population of citizens

We next analyse deliberation in a population of voters akin to a citizen assembly. We consider the simple but most relevant case where the population is divided into two groups, each with its own thinking frame about the voting issue. The ICB example is a suitable one as it relates to quite well-established ideologies: a left leaning social and environmental ideology ($L=\left(L_{1},L_{2}\right)$), and a right leaning conservative libertarian ideology ($R=\left(R_{1},R_{2}\right)$). We assume that $L_{1}$ and $R_{1}$ give rise to a yes vote, while $L_{2}$ and $R_{2}$ give rise to a no vote. Hence, the two consensual states are $\left(L_{1},R_{1}\right)$ and $\left(L_{2},R_{2}\right)$. To ease comparison with the previous section we define $\text{Tr}(L_{1}R_{1})=\text{Tr}(L_{2}R_{2})=x$.

We first establish a simple lemma:

**Lemma 1**

*Starting from consensus, one round of deliberation between two citizens with distinct perspectives leads to disagreement with positive probability.*

*Proof*. Consider starting from the consensus state $\left(L_{1},R_{1}\right)$ and letting the R-citizen probe the L perspective, we are back in $\left(L_{1},R_{1}\right)$ with probability $\text{Tr}(R_{1}L_{1})\text{Tr}\left(L_{1}R_{1}\right)+\text{Tr}(R_{1}L_{2})\text{Tr}\left(L_{2}R_{1}\right)=x^{2}+(1-x{)}^{2}<1$, so consensus is lost with positive probability for x∉{0,1}*,* that is, when the two perspectives are distinct from each other.∎

Lemma 1 illustrates that when two citizens agree on voting, it can be harmful for the existing consensus to let them explore an alternative perspective, as it may trigger a change in opinion. As we show below, this feature has implications for the optimal strategy of the facilitator in the case of an assembly of citizens. We note that this observation is consistent with the criticism that political interaction can have a detrimental impact on citizens’ beliefs and preferences [4,41]^27^.

Let the two groups be of size l, respectively, r. We shall assume that in each group the citizens have determined themselves in their own frame among the two possible opinions. Hence, among the L-citizens, $l_{1}$ are in the $L_{1}$ state, and $l_{2}=l-l_{1}$ in the $L_{2}$ state. We define similarly $r_{1},r_{2}$ for the R-citizens. Note that in contrast with the previous analysis (with only two citizens), there can now be disagreement among citizens sharing the same perspective. We assume, without loss of generality, that $l_{1}+r_{1}>l_{2}+r_{2}$, so that there is an initial majority for the yes vote. The objective of the facilitator is to maximize the score in the current round. The first step is to select a *projected consensus state*, defined as the consensus state that the facilitator aims at maximizing support (score) for. The next step is to select the citizens who will be invited to probe a perspective. By lemma 1, we know that only citizens disagreeing with the projected consensus should be invited to be active. Otherwise, the facilitator risks losing some support for his projected consensus. In general, there are citizens who disagree in both L and R perspective. The probability that one citizen switches her voting intentions when confronted with the alternative thinking frame is equal to 2x(1−x). The gains in terms of score from a deliberation round are therefore proportional to the size of the group (of disagreeing citizens) selected for probing. As we shall, counterintuitively, see this may imply selecting the minority consensual state as the projected consensus. We have the following proposition:

**Proposition 2**

*Starting from disagreement within and/or between two groups, the optimal strategy for the facilitator in each round entails:*

(i) *Projected consensus state: select as consensus state the state associated with the largest expected score given the optimal choice of target group and perspective. It may be optimal to aim at overturning the initial majority;*

(ii)* Selective targeting: Select the largest group of citizens (either in group* L *or* R*) who disagree with the projected consensus for probing. The remaining citizens from that perspective group refrain from probing;*

(iii) *Starting from a nearly even distribution of voting intentions in the two groups, deliberation can deliver an expected majority of 3/4 of the population after two rounds.*

*Proof*. (Proposition 2(i)). We know from proposition 1 that the probability of opinion change is 2x(1−x)=Δ. The expected change is thus $\Delta l_{i}$ (or $\Delta r_{j}$) where i,j=1,2 depending on the group invited for probing. Consider a case where $(l_{1}+r_{1})>\left(l_{2}+r_{2}\right)$, so, the majority supports yes. Then whenever $l_{1}+r_{1}+\Delta \;\max(l_{2},r_{2})<(l_{2}+r_{2})+\Delta \;\max(l_{1},r_{1})$, that is,$(l_{2}+r_{2})-(l_{1}+r_{1})>\Delta [\max(l_{2},r_{2})-\max(l_{1},r_{1})]$, the optimal projected state corresponds to the no (minority) vote. When the inequality goes the other way, the standing majority is the optimal projected consensus state.

(Proposition 2(ii)). Given proposition 2(i), the largest group of citizens disagreeing with the projected consensus is identified. Because of lemma 1, no other citizen from that perspective group should be invited to probe.

(Proposition 2(iii). Let $l_{1}=l_{2}=l/2$ and similarly $r_{1}=r_{2}=l/2$ with Δ=1/2. Any of the two consensus states can be reached with the support of a population of size l/2+r/2+l/4+r/4=(3/4)(l+r).∎

The results in proposition 2 invite multiple remarks. First, in each period, the projected consensus state is not necessarily the standing majority. This is because we are dealing with a population of citizens, so the size of the group that could switch opinion matters. While the majoritarian consensual state holds an advantage, optimal deliberation can favour the minority position. This is quite remarkable because the result applies already when the facilitator is ‘myopic’, i.e. maximizes the score in each round. This result is a nice property of the deliberation procedure, as it implies that it gives a fair chance to the minority.^28^ We illustrate the point in a numerical example:

**Example**

Consider the following initial situation with Δ=1/2: $l_{1}=60,\;l_{2}=40$ and $r_{1}=20,\;r_{2}=35$ implying $l_{1}+r_{1}=80>l_{2}+r_{2}=75$, so the standing majority is $l_{1}+r_{1}$. If the facilitator selects $L_{2}R_{2}$ as the projected consensus state, after one round the expected score is $l_{2}+r_{2}+\Delta l_{1}=105,$ which is larger than the score he can reach with the standing majority ($l_{1}+r_{1}+\Delta l_{2}=100$), so the facilitator optimally leads deliberations to overturn the initial majority.

A second important remark is that the optimal strategy actualizes the distinction between active and passive citizens. Because of lemma 1, when both opinions are present in a perspective group, it is optimal for the facilitator to proceed selectively. Those from the targeted perspective group already agreeing with the projected consensus state should refrain from probing, as they could change their mind and reduce the score. This means that the optimal procedure implies to some extent the unequal treatment of citizens, which is unfortunate from a democratic point of view. Note, however, that full publicity of debates can be preserved because, with contextual opinions, only the operation of probing can induce change. Simply listening to an argument without making the effort of thinking in the terms of the alternative perspective has no effect on opinions.

Our analysis of the population case provides an interesting rationale (and guiding principles) for the practice of parallel working groups encountered in real-life deliberation. We could say that it responds to the awareness that there exist risks that deliberation ‘breeds confusion’ in people’s minds. Returning to the illustration, we could have a single round with two parallel working groups, one made up of $l_{1}$ and the other of $r_{1}$.^29^ Such a procedure would prevent unwanted opinion switch when citizens make probing operations without being invited to do so. Subsequent rounds could appeal to experts, exposing arguments from a maximally uncorrelated perspective to remaining disagreeing citizens. Obviously, this accelerates the process towards consensus.

We thus find that our results from proposition 1 carry over to a population of citizens. Unexpectedly, we also find that the initial distribution of opinions does not fully determine the outcome of voting which is a nice property. Less attractive from a democratic point of view is that citizens are not treated equally. We suggest that the necessary discrimination can be implemented less controversially when the probing operations are carried out in well-composed working groups.

## 5. Concluding remarks

In this paper, we have developed a formal approach to deliberation based on the behavioural premises and the mathematical formalism of quantum cognition. Deliberation is formulated in the context of complete information as a structured communication process managed by a facilitator with the aim of maximizing the probability for consensus in a binary collective choice problem. The process includes a sequence of rounds in which some citizen (or expert) develops an argument belonging to some perspective, and other citizens are invited by the facilitator to probe that perspective. Probing involves a true mental experience for the citizens. They have to think in terms of the probed perspective and decide how they position themselves.

A first central result is that the incompatibility of perspectives, that is, the diversity of viewpoints among citizens, is what permits opinions to evolve. Our second central result is that the correlation between perspectives is the key property that determines the pace of evolution towards consensus. In the two-citizens case, starting from disagreement, the highest probability for consensus is achieved when the citizens’ perspectives are maximally uncorrelated. The results generalize to populations of citizens where the facilitator’s strategy involves some inequality in treatment: only a selected subset of citizens is invited to perform the probing operation, while the others simply listen. The population case reveals that optimal deliberation may overturn the initial majority, which gives some chance to the initial minority option.

From a purely formal perspective, equivalent results to those presented in this paper could be obtained without appealing to the quantum-like formalism. One could build a tailored classical model involving random jumps between opinion states when probing different thinking frames and reproduce the results. However, such a model would appear rather arbitrary and lack a fundamental cognitive motivation and interpretation, as opposed to our quantum-like cognitive model which displays such random opinion changes as a built-in feature.^30^

Our analysis delivers a transformative power of deliberation, going beyond Bayesian updating. In our model, people go through real (mental) experiences (probing) that transform their opinion. They are in a state of complete information, but they learn how the issue at stake can be looked at from equally valid alternative perspectives and how these relate to their own. This resonates with the normative imperatives often put forward by deliberative democrats that deliberation requires engagement and fosters the respect for others as a practical school of democracy. In our context, this is captured by the willingness and actual experience of ‘putting oneself in someone else’s shoes’. Our formal result on the constructive value of diversity is truly novel. It echoes Bohman [17] who views the transformative power as intimately linked with the diversity of perspectives. Our approach allows characterizing the determinant role of the facilitator. This is consistent with real-life experiments with citizen assemblies, which recognize a central role for facilitators. Not surprisingly, the optimal strategy of the facilitator features a tension between equity and efficiency, translated into the recommendation for some citizens to be active while others should rather remain passive. Interestingly, this result can provide guiding principles for setting up working groups in parallel sessions. Finally and importantly, our analysis reveals a value of deliberation in terms of improving consensus not based on improved information. Instead, our results exclusively appeal to the intrinsic contextuality of opinions, i.e. their inseparability from the thinking frame in which they are formulated.

We wish to emphasize that the model depicts a very stylized situation aimed at providing insights relying on rigorous (and transparent) formal results. The flip side is limitations in terms of immediate practical implications. Many of those are related to the assumptions. Clearly, not all citizens maybe be willing to follow the facilitator’s recommendations (assumption 4). In particular, some may refuse to explore alternative perspectives, in which case their opinion does not evolve. Others may explore perspectives when asked to remain passive, which triggers unwanted opinion change. Next, it is not realistic to assume the facilitator’s omniscience and unlimited access to suitable experts’ perspectives (assumption 5). Violations of these assumptions reduce the efficiency of the proposed deliberation protocol in terms of speed of convergence. The qualitative results and the basic insights from the analysis remain in force, however. We are currently planning an experiment to test the protocol to gain knowledge about issues related to its practical implementation. A detailed description of that project is out of the scope of the present paper.

## Data Availability

This article has no additional data.

## Footnotes

^1^
Deliberative institutional experimentation is flourishing throughout the world (a catalogue is available at https://participedia.net/).

^2^
Thomas [11, p. 245] writes ‘Their thesis is that democratic decision-making ought to be grounded in a substantial process of public deliberation, wherein arguments for and against laws and policies are given in terms of whether they advance the common good of citizens and the justice of the political society’. Rawls’s idea of ‘public reason’ is on the same line.

^3^
See Arendt (2014) ‘ Qu'est-ce que la politique’ pp. 63–64 Edition du Seuil [18].

^4^
This implies that we are not addressing the impossibility theorems of collective choice (see e.g. Arrow [23]), which apply when the choice set includes at least three options.

^5^
Perspectives A and B are said to be maximally uncorrelated whenever a citizen's opinion in perspective A has no predictive power for her opinion in perspective B.

^6^
The picture is only illustrative. It shows how two distinct and incompatible mental pictures can be correct and each provide a complete characterization, since both give sense to all the details. It does not feature all the quantum properties that we appeal to in the paper, however. Similar pictures (e.g. Necker cube) have been studied in terms of the quantum properties of bistable perception ( e.g. [30]). For a history of this picture, see https://en.wikipedia.org/wiki/Rabbit

^7^
In particular, Bohr was influenced by the psychology and philosophy of knowledge of Harald Höffding ([31] and the Introduction in [32] for an insightful discussion).

^8^
It parallels the complementarity of incompatible properties for subatomic particles, e.g. the spin along different directions

^9^
The approach is an abstract way of capturing the fact that experiencing another perspective may have neurological, emotional and other impacts with consequences for opinion.

^10^
Note that quantifying correlations is an empirical issue in physics as well.

^11^
A quantum lottery is a bet on a quantum measurement.

^12^
Physicists work with the complex field. For simplicity, and because it is sufficient to establish our results, we shall work with the real field ℝ, although everything goes with minor changes for the complex case. This also means that for all relevant purposes our Hilbert space boils down to a Euclidian space.

^13^
In Dirac's notation, |ψ⟩ denotes a column vector, and ⟨ψ| the row vector which here is the transpose of |ψ⟩. The projector $P_{\psi }$ is an operator, represented as a n×n matrix, such that $P^{2}=P$.

^14^
As opposed to more general mixed states, not considered in this work. Pure state are complete information states, but because of intrinsic indeterminacy, the values along alternative (incompatible) perspectives can never be sorted out simultaneously, they remain stochastic.

^15^
Notice that the utility function is different for each citizen j∈Ω.

^16^
This is more demanding than simply deciding to agree or reject the presented opinion. Indeed, if the argument is rejected, the revised state would not be an eigenstate of the perspective under exploration but a superposition of states. Such a measurement operation would be coarse while we focus on complete measurements.

^17^
Passive citizens do not make the effort to find out how they think in the terms of the proposed perspective.

^18^
How to safeguard against possible manipulations by the facilitator is a subject of great importance that we leave for future research.

^19^
Note that the facilitator is not analysed in quantum terms. He could be replaced by an algorithm that maximizes the objective function by choosing which perspective should be probed by whom in each round.

^20^
This is a feature of the mathematical formalism of QM—all relevant history is encapsulated in the current state.

^21^
This is not fully without loss of generality. We show in [40] that under some circumstances, it can be optimal to deviate from this short-sighted strategy.

^22^
This is most natural since they are expected to be invited to expose their opinion.

^23^
Notice that the formalism of QM would allow in general as (pure) states any projector acting on the Hilbert space ${\mathbb{R}}^{4}$. Such generic, so-called *entangled states*, contain non-classical correlations between Alice's and Bob's states. In our protocol, only *product states* of the form ${\text{P}}_{\psi }^{t}=(P_{\psi_{A}},P_{\psi_{B}})$ are involved. We focus on this simpler case and write ${\text{P}}_{\psi }^{t}=(P_{\psi_{A}},P_{\psi_{B}}).$

^24^
Note that once voting intentions have been identified for each opinion state, the analysis can proceed without reference to utility values.

^25^
In the quantum information literature, such A and B define *mutually unbiased bases*.

^26^
Two perspectives are formally the same when they are fully correlated. They may have different labels, however.

^27^
Janis introduced the term ‘groupthink’ to capture the possible downside of deliberation (among high-level political decision-makers). Groupthink reinforces biases, pushes towards conformity with the dominating views, blinds for arguments going against the majority etc. The idea here is that a person in isolation may be confused after having been confronted with arguments in deliberation because she is the victim of groupthink.

^28^
In our general paper, we show that overturning the initial majority can also be optimal when the facilitator maximizes consensus probability over several rounds [40].

^29^
Note that $r_{2}$ citizens would be free to join the first group and $l_{2}$ the second working group. since they would be probing their own perspective, that would not affect their opinion.

^30^
In [42], the authors investigate a general measurement theory and show that if the interaction between systems and measurement instruments satisfies some natural conditions, the state space is endowed with the structure of an atomistic orthomodular orthospace and that the states are realized as probability measures on the state space. They identify the additional axioms that lead (almost) all the way to the Hilbert space model of QM that we use in quantum cognition. All the axioms are discussed in terms of their relevance for social sciences.
